# Comparing methods for constructing and representing human pangenome graphs

**DOI:** 10.1186/s13059-023-03098-2

**Published:** 2023-11-30

**Authors:** Francesco Andreace, Pierre Lechat, Yoann Dufresne, Rayan Chikhi

**Affiliations:** 1 Department of Computational Biology, Institut Pasteur, Université Paris Cité, Paris, F-75015 France; 2 Sorbonne Université, Collège doctoral, F-75005 Paris, France; 3 Bioinformatics and Biostatistics Hub, Institut Pasteur, Université de Paris, F-75015 Paris, France

**Keywords:** Pangenomics, de Bruijn graphs, Variation graphs, Sequence analysis, Algorithms

## Abstract

**Background:**

As a single reference genome cannot possibly represent all the variation present across human individuals, pangenome graphs have been introduced to incorporate population diversity within a wide range of genomic analyses. Several data structures have been proposed for representing collections of genomes as pangenomes, in particular graphs.

**Results:**

In this work, we collect all publicly available high-quality human haplotypes and construct the largest human pangenome graphs to date, incorporating 52 individuals in addition to two synthetic references (CHM13 and GRCh38). We build variation graphs and de Bruijn graphs of this collection using five of the state-of-the-art tools: Bifrost, mdbg, Minigraph, Minigraph-Cactus and pggb. We examine differences in the way each of these tools represents variations between input sequences, both in terms of overall graph structure and representation of specific genetic loci.

**Conclusion:**

This work sheds light on key differences between pangenome graph representations, informing end-users on how to select the most appropriate graph type for their application.

**Supplementary Information:**

The online version contains supplementary material available at 10.1186/s13059-023-03098-2.

## Background

In recent years, the majority of studies on human genetics have been conducted on the basis of comparing new samples against a single, standard reference sequence. This reference sequence is a linear succession of nucleotides that acts as a blueprint of the human genome. It is routinely used to align raw sequencing data to it in order to find variations between genomes, e.g., single-nucleotide polymorphisms (SNPs), insertions, or deletions (indels). It also is the backbone of the UCSC Genome Browser [1] which enables inspection of genomic and epigenomic features. Despite updates that have improved the quality of the human reference sequence in the last two decades, its linear form severely limits the ability to capture population genetic diversity. For instance, the locations of frequently observed structural variations cannot be easily integrated into a linear reference. To see this, consider the difficulty of designing a suitable coordinate system in the presence of (possibly nested) structural variants. Having a single genome as a reference sequence also introduces an observational bias towards the chosen alleles that were integrated into that sequence, negatively impacting many primary analyses such as reads mapping, variant calling, genotyping, and haplotype phasing. As a result, our ability to precisely characterize structural variants, SNPs, and small indels is limited [2–4]. The GRCh38 human reference genome is estimated to miss up to 10% of our species genetic information [5].

Improvements in sequencing data quality and length, as well as genome assembly methods, are providing a fast-expanding collection of haplotype-resolved human genome assemblies. If adequately combined together, these high-quality individual genomes may offer a powerful alternative to the linear reference. There now is an active line of research on pangenomes, i.e., data structures that represent a collection of genomic sequences to be analyzed jointly or to form a reference [3, 6]. Pangenome-based approaches have been shown to improve biological analyses. Pangenomes are at the basis of bioinformatics tools that perform high-quality short read mapping [4], genotyping of SNPs, indels, and SVs [7], RNA-seq mapping [8]; de novo variant calling [2]; to store, compress and retrieve high-quality genomes [9]; to condensate all the information from a high number of genomes to then visualize specific regions or perform ad-hoc analysis, particularly on complex loci, SVs, and tandem repeats [8]. These results pave the way for new applications, e.g., genome-wide association studies, where more precise identification of variants can improve the scope of genetic studies in aging, human diseases, and cancer [3, 6].

Several pangenomic data structures have been proposed: multiple sequence alignments, de Bruijn graphs, cyclic and acyclic variation graphs, and haplotype-centric models that use the Burrows-Wheeler transform  [3]. Each of these approaches aims to represent a collection of genomic sequences in an efficient way, to store, visualize, and retrieve differences of interest between the considered genomes. Graph-based pangenome data structures, such as the de Bruijn graph and the variation graph, appear so far to be the most advanced in their ability to handle large amounts of input data. They are capable of representing tens to hundreds of human haplotypes simultaneously. Variations graphs use a sequence graph and a list of paths to store input haplotypes, while de Bruijn graphs store all haplotype *k*-mers annotated by their haplotype(s) of origin.

Scaling pangenome graph data structures to store hundreds of genomes is a challenge that requires significant computational resources and engineering efforts. Many software tools have been created, here we briefly describe major ones. Pantools [10] and Bifrost [11] are two methods that have been developed to generate pangenomes for analysis on large collections of genomes, mostly for applications in phylogenetics and bacterial genomics. The PanGenome Graph Builder (pggb) [12], Minigraph-Cactus and TwoPaCo [13] are methods for building general-purpose pangenome graphs. Minigraph [14] builds a particular type of pangenome graph by aligning sequences in an iterative way to a reference template. Minimizer-space de Bruijn graphs (mdbg) [15] are variants of de Bruijn graphs that can efficiently represent very large collections of bacterial pangenomes (e.g., 600,000 bacteria). vg [2] builds variation graphs from a reference sequence and a variant calling file (vcf) that contains a list of variations from it.

Many human pangenomes have been generated, e.g. using Pantools [10] (7 genomes), Minigraph [14] (94 haplotypes), Minigraph-Cactus [16, 17] and pggb [8] (94 single chromosomes), and TwoPaCo [13] (100 simulated genomes). Lastly, a draft version of a human reference pangenome constructed using pggb and the Minigraph-Cactus pipeline has appeared in a very recent article from the Human Pangenome Reference Consortium [8]. These pangenomes are still limited by some factors: at the present moment, the number of high-quality haplotype assemblies is still low, even if it is expected to grow in the future; the vcf files containing variation are limited in terms of bias, type of variation or number of samples; the population representation, even if opened up in recent years to more ethnicities, is still affected by sampling bias.

## Results

In this article, we provide a comprehensive view of whole-genome human pangenomics through the lens of five methods that each implement a different graph data structure: Bifrost, mdbg, Minigraph, Minigraph-Cactus, and pggb. We examine several features of pangenome graphs, in particular their scalability and how they represent genetic diversity. To this end, we collected all publicly available high-quality human haplotypes and attempted to construct pangenomes of various complexity with each selected tool. Although vg has been widely used as the basis of relevant pangenome-based discoveries, for example on fast and accurate short read mapping [4], we decided to not consider it in our analysis for two main reasons: the bias introduced by the reference sequence that is used as the backbone of the graph (and associated to the vcf) together with the limited capacity of this method to integrate structural variations from many genomes. We believe both aspects are drivers of the use of pangenome graphs.

### Scalability and characteristics of pangenome graph construction tools

We ran the above five tools on three datasets consisting of 2, 10, and 104 human haplotypes, respectively (Table 3). We compared the computational performance of construction algorithms as well as characteristics of the produced pangenome graphs. The goal is to assess the ability of each method to scale to data available in the near future, i.e., thousands or even millions of human genomes [5].

The performance of each tool is evaluated in terms of running time, peak memory, disk space required by the output data structure (graph and annotations). We also compared the number of nodes, edges and connected components as indicators of the complexity of the graph. Results are displayed in Table 1.

In terms of running time, mdbg is two orders of magnitude faster than other tools on all considered datasets, taking around two minutes on the H2 dataset and half an hour on H104. Bifrost is the second fastest on H104 (18 hours), and Minigraph is the second fastest on H2 (8 minutes). Minigraph-Cactus takes one order of magnitude more time than Minigraph. We could not obtain graphs for pggb and Minigraph-Cactus on H104 as for the first the execution did not finish after 2 weeks and the second returned an error.

In terms of memory usage, mdbg consistently uses less than half the memory of other tools (31 GB on H104), followed by Minigraph (61 GB on H104). On H2 all tools used between 8 and 66 GB of memory.

All tools used reasonable disk space to store the resulting graph, $\le 12$ GB for H10 and $\le 38$ GB for H104. Although Minigraph-Cactus and pggb retain all variations and are the only two tools able to reconstruct the input haplotypes directly from the graph, they are the second and third most efficient in term of disk space (for Minigraph-Cactus, 3.6 GB on H2 and 7 GB on H10). While Bifrost and Minigraph perform all computation in memory, pggb, Minigraph-Cactus, and mdbg store intermediate files on disk, taking comparable space to the input size (up to 3× for Minigraph-Cactus).

### Different tools yield different pangenome graphs topologies

Graph metrics such as the number of nodes, edges and connected components provide useful insights on the level of detail of the represented variations and on the complexity and accessibility of the information inside the pangenome.

The number of graph nodes varies between 17,000 and 11 millions for the H2 dataset across all tools. In all cases, the number of nodes is at least 3 orders of magnitude smaller than the number of bases in the haplotypes, indicating that pangenome graphs are effective at compressing linear parts of the haplotypes. Tools which discard variations (Minigraph and mdbg) yield in the order of $10^4$–$10^5$ nodes across all datasets, while tools which retain all variation (Bifrost, Minigraph-Cactus and pggb) yield in the order of $10^6$–$10^7$ nodes. In all cases going from the H10 dataset to the H104 dataset increases the number of nodes by 5x, indicating that graph complexity grows sublinearly with the number of added haplotypes.

The number of connected components varies between 2 and 1402 across all methods and datasets, and the number of large components (i.e., those with more than 1% of total base pairs) varies between 1 and 30. If chromosomes were separated perfectly, pangenome graphs should contain exactly 24 connected components (one per nuclear chromosome, excluding mitochondria). Minigraph produces 24 large connected components as the number of chromosomes in the reference CHM13 v2.0 (25 including mitochondria). Bifrost and Minigraph-Cactus yield graphs with less than 25 connected components while mdbg and pggb have more than 25. In the Bifrost dBG, the vast majority of sequences (> 99.99%) are in a single giant component, as chromosomes are joined because they share common *k*-mers. In mdbg, such joining does not occur on dataset H2, which has 24 large enough components (each containing > 1% of bases), possibly due to the absence of long and similar enough regions between chromosomes. Minigraph does not map any mitochondrial sequence from the input haplotypes to the reference, while they do get included into Minigraph-Cactus graphs.

**Fig. 1:**
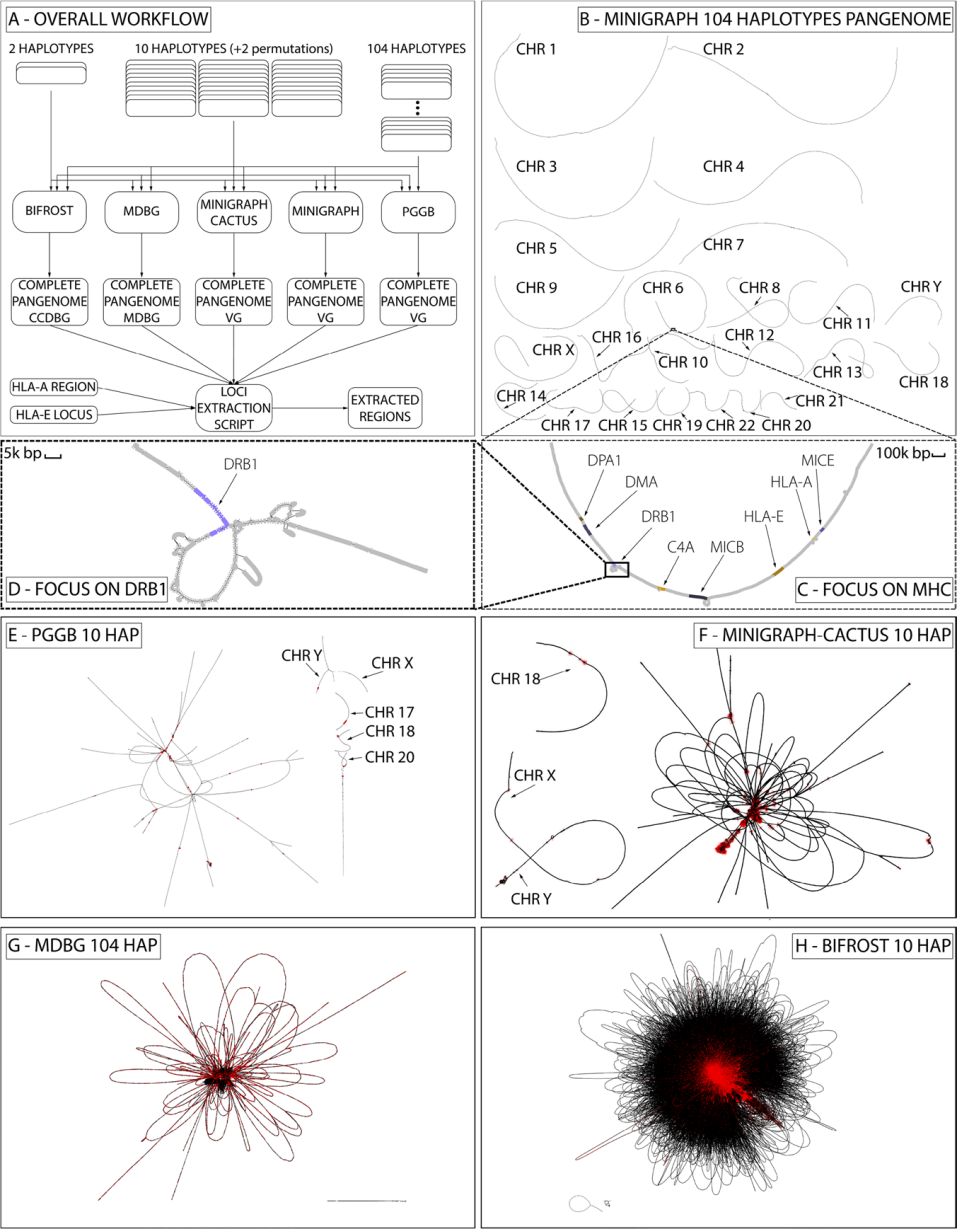
The complete pangenome construction scheme and visualization. **A** The overall workflow, using 5 different tools on 3 different datasets; **B** complete 104 haplotypes variation graph built by Minigraph; **C** focus on part of HLA (MHC) region in chromosome 6 from panel **B**; **D** focus on DRB1-5 locus of HLA from panel C; **E**, complete 10 haplotypes variation graph built with pggb; **F** 10 haplotypes variation graph built with Minigraph-Cactus; **G** 104 haplotypes pangenome mdbg; **H** 10 haplotypes Bifrost dBG. All graphs except those produced by Minigraph have been simplified using gfatools and rendered using Bandage. VG is for variation graph

Even if it is common practice to analyze pangenomes chromosome by chromosome [8, 17], in this analysis we purposely used entire genomes as input instead. This was done for two reasons: i) to highlight the scalability of the tools, and ii) because separating chromosomes prevents the identification of inter-chromosomal inversions, translocations, and transposable elements, even if most of the generated inter-chromosomal events are probably alignment artifacts. The effects of this choice can be seen in the pggb and the Minigraph-Cactus H10 variation graphs of Fig. 1. In the pggb graph 19 chromosomes are linked into a single giant component, while chromosomes 17, 18, 20, X, and Y are in other large components. This giant component consists of 25 M nodes that contain 83% of the total basepairs. The remaining 859 components represent only 4.7% of the total bases due to small sequences in the input haplotypes. In the Minigraph-Cactus graph all chromosomes are linked into a single giant component except chromosome 18 that is in a separate component, and the sexual chromosomes (X and Y) that are connected together into another component.

**Table 1:** Time, memory, final disk space, nodes, edges, total connected components and connected components with more than 1% of base pairs comparison of Bifrost, mdbg, pggb, Minigraph and Minigraph-Cactus for different number of haplotypes in input. Minigraph-Cactus times include the Minigraph graph construction step. pggb was not able to complete its execution on the largest dataset in more than 2 weeks thus it is not considered. Minigraph-Cactus failed to compute the 104 HAP dataset

Haplotypes	Metric	Bifrost	pggb	Minigraph	Minigraph-Cactus	mdbg
2	Time (hh:mm:ss)	1:21:25	15:45:30	00:08:33	3:11:59	00:02:38
	Memory (GB)	53	24	38	66	8
	Disk space (GB)	4.8	4.3	2.9	3.6	4.4
	Nodes	9482 k	8492 k	34 k	10,851 k	17 k
	Edges	13,108 k	11,503 k	48 k	14,702 k	23 k
	Conn comp	2	1402	25	4	174
	Conn comp > 1% bp	1	30	24	4	24
10	Time (hh:mm:ss)	2:27:29	117:08:09	2:03:29	15:57:05	00:05:46
	Memory (GB)	102	71	49	154	18
	Disk space (GB)	12	7.6	2.9	7	9.7
	Nodes	27,468 k	29,315 k	133 k	37,767 k	67 k
	Edges	37,584 k	40,282 k	190 k	51,595 k	93 k
	Conn comp	3	864	25	3	40
	Conn comp > 1% bp	1	5	24	3	1
104	Time (hh:mm:ss)	18:38:28	—	46:22:00	—	00:31:38
	Memory (GB)	122	—	61	—	39
	Disk space (GB)	29.4	—	3.2	—	38
	Nodes	106,339 k	—	632 k	—	270 k
	Edges	293,839 k	—	912 k	—	396 k
	Conn comp	17	—	25	—	1097
	Conn comp > 1% bp	1	—	24	—	1

### Interpretation of variation in pangenome graphs: focus on two HLA loci

The ability to detect and annotate variations among input haplotypes defines the scope of each pangenome graph construction method. Previous work [18] recommends to build graphs on a specific loci rather than the entire genome for the purpose of i) identifying genomic diversity and ii) mapping raw reads to divergent regions, specifically difficult-to-map repeats. Here we evaluate how pangenomes built from entire haplotypes represent specific biologically relevant loci.

#### Extraction of HLA-E and a complex HLA region from complete pangenome graphs

We extracted from complete pangenomes the regions corresponding to two loci of the Human Leukocyte Antigen complex, also known as HLA. These regions are highly medically relevant as they contain many disease-associated variants [19]. The first locus is the HLA-E gene, that is part of the nonclassical class I region genes, spanning 4.8 kbp, and is relatively conserved across populations. It has been shown to have a significant association with hospitalization and ICU admission as a result of COVID-19 infection [20]. The second is an HLA complex region comprising the HLA-A gene, part of the classical, highly polymorphic class I region. It is around 58 kbp long and contains the HLA-U, HLA-K, HLA-H, and HCG4B genes. We extracted these two regions from pangenome graphs using a custom script that yields a subgraph corresponding to a given set of sequences and their variation. The script uses a different recommended method for each of the pangenome graph types. In a nutshell, we extracted regions using exact coordinates when possible and resorted to sequence-to-graph alignment otherwise (see Appendix “Loci extraction method” section for details). While on variation graphs and mDBGs nearby nodes of an aligned region correspond to variations of the locus, this is not always true for standard dBGs. Extracting accurate and complete loci representation is an unsolved challenge for dBGs.

#### HLA-E: a low complexity region 

**Fig. 2:**
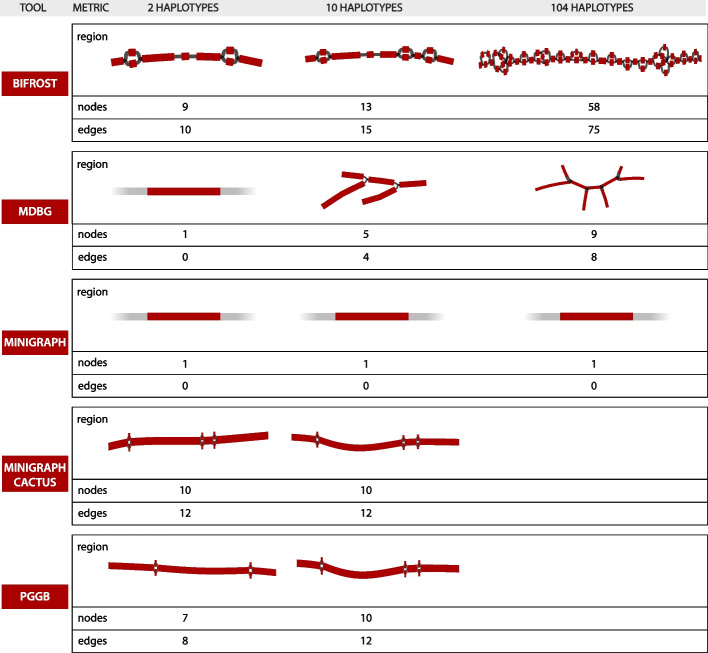
Representations of the HLA-E locus by five graph construction methods over three increasing large human pangenomes. Nodes highlighted in red contain part of the locus sequence. The number of nodes and edges displayed below each graph concerns the whole subgraph (both red and gray nodes). Minigraph, on H2, H10, and H104, and mdbg, on H2, have only a portion of one node highlighted since the 4.8 kbp region is contained inside a single, long node

Figure 2 shows how the different tools represent HLA-E over datasets H2, H10, and H104. As expected, Minigraph does not detect any variation, since the SNPs that characterize the region are too small to be considered in the construction steps of their algorithm. pggb, on the contrary, has 2 SNPs in H2 and 3 in H10. Bifrost detects the same SNPs as pggb in H2 and H10. Both of them represent the exact same variations and render the same haplotype paths. mdbg captures the heterozygosity of a large region containing the HLA-E locus as the number of samples grows. As the mdbg graph is built in minimizer space, nodes represent long genomic segments (in the order of hundreds of thousand of base-pairs). In H10 and H104, the minimizer-space representations of the haplotypes are identical; however, differences in flanking regions of the graph create variations that are captured in extra nodes that are also extracted in this region. On H2, Minigraph-Cactus detects 3 variations as the dataset used is different, containing the CHM13 reference and just one haplotype of HG006 (as in Minigraph), as discussed in the “Datasets and haplotypes collection” section.

Figure 2 also illustrates how pangenome complexity grows with the number of genomes: the Bifrost H104 subgraph has the most variation across all methods, highlighting that dBGs represent variations exhaustively in large graphs. On the other hand, pggb has the most straightforward method for extracting subgraphs, and also represents variants exhaustively in datasets H2 and H10, but could not scale to the H104 dataset.

#### HLA complex locus: high complexity region 

**Fig. 3:**
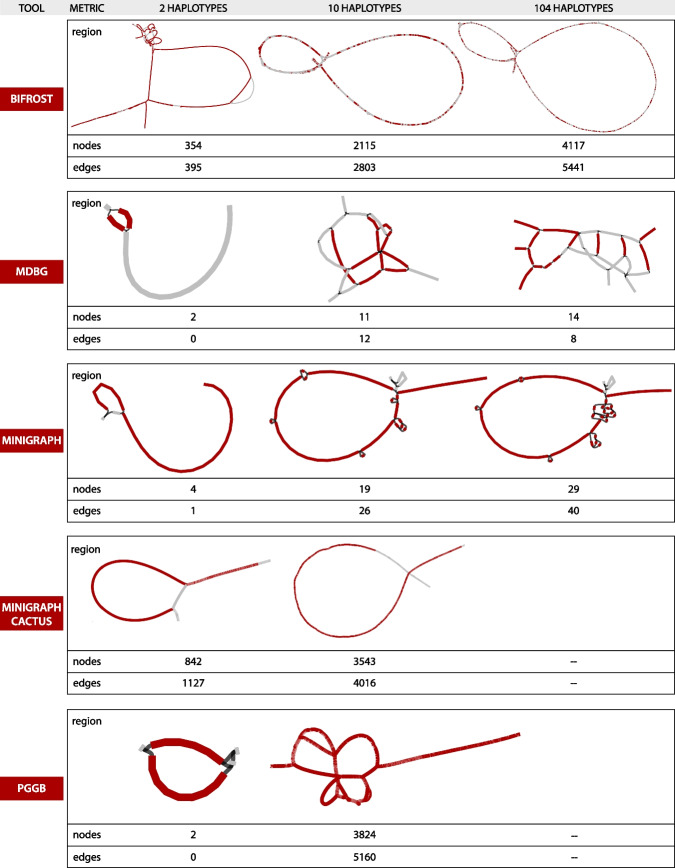
Representations of the complex HLA region by five graph construction methods over three increasing large human pangenomes. See caption of Fig. 2 for details

Figure 3 is the counterpart of Fig. 2 for the complex locus part. In this case, the overall interpretability of the region is more challenging, as the number and the structure of the variations is different than in HLA-E. It is also more difficult to compare across tools. Base-level variations, e.g., SNPs, are not visually recognizable in Fig. 3 in the methods that retain them (i.e., pggb, Minigraph-Cactus, and Bifrost) due to the large sizes of graphs.

There are notable differences in how tools represent the variation, which is well-illustrated in the H2 dataset. While Minigraph renders H2 as a single sequence plus a large structural variant (SV) of $\approx$ 52 kbp, pggb separates it into two paths that differ by $\approx$ 54 kbp in length. Bifrost represents a detailed bubble that contains many variations inside each path. In mdbg, even extracting the complete locus is a challenge as many of the subgraph nodes were not selected by our procedure. Minigraph-Cactus adds base level divergences between haplotypes on top of Minigraph SV graph.

These differences between representations are further accentuated in the H10 dataset. For it, pggb tends to separate the haplotypes into different paths, Bifrost renders consistently the same compacted representation and Minigraph neglects most of the small differences but is able to display accurately the general picture, and Minigraph-Cactus, as in H2, adds small variations on top of Minigraph structure.

### Uncovering characteristics of graphical pangenome tools

The data structures generated by pangenome building tools are expected to facilitate comparisons between the input genomes. In addition, pangenome graphs should be stored in such a way to be easily used by downstream applications. We identify 8 important features for pangenome graph construction tools: (i) stability, (ii) editability, (iii) accessibility by downstream applications, (iv) haplotype compression performance, (v) ease of visualization, (vi) quality of metadata and annotation. Two other but important features, scalability, and interpretability of produced graphs, were already discussed in the “Scalability and characteristics of pangenome graph construction tools” and “Interpretation of variation in pangenome graphs: focus on two HLA loci” sections. Table 2 summarizes some of the following considerations on the relative strength of the tools.

#### Editability and dynamic updates

As more high quality assemblies will be generated in the near future, haplotypes may be added to a pangenome, or replaced by improved versions. Updating an existing data structure instead of rebuilding it from scratch is both computationally and energetically efficient. However, many succinct data structures currently used in pangenome representation are static, i.e., cannot be updated. Some methods allow a restricted set of editing operations. Minigraph allows to add new haplotypes on top of an already built graph. Bifrost provides C++ APIs to add or remove (sub-)sequences, *k*-mers and colors from the ccdBG. pggb, using odgi [21], allows specific operations that delete and modify nodes and edges and add and modify paths through the graph. As Minigraph-Cactus can be opened with odgi, it supports the same operations as pggb. The current mdbg implementation uses a dynamic hash table, but does not expose an interface that supports updates.

#### Stability 

Counter-intuitively, a pangenome graph construction tool may in some cases generate different outputs when executed multiple times with the same haplotypes as input. This *unstability* could be due to a permutation in the order of the sequences given as input, or non-determinism in the construction algorithm. Yet in order to facilitate the reproducibility of results, pangenome building tools should generate an unchanged output from the same set of input sequences, independently of the particular run or the order in which these are given. We performed two tests to evaluate tool stability: (i) we run the tools 3 times using as input the same H10 dataset and ii) we run the tools twice on shuffled input sequences, i.e., changing the order of the haplotypes of H10.

Bifrost and mdbg constructed exactly the same pangenome on every test, as by definition, de Bruijn graphs are stable. Minigraph generates identical graphs on identical inputs, but generates slightly different graphs when the input is permuted. Indeed the construction algorithm of Minigraph is order-sensitive as it augments the existing graph structure by aligning the next given haplotype to it and adding divergent sequences. Minigraph-Cactus generates slightly different graphs on identical input. pggb generated slightly different graphs while maintaining the same haplotype sequences in the paths. The overall representation of the input genomes is therefore preserved, while the topology of the variation graph varies. The first two of the three phases of the pggb pipeline (all-vs-all alignment and graph imputation) produce the same result on different runs with the same input but differences arise when the order of the input haplotypes changes. Most of the differences in the graph topology are thus due to the final smoothing steps.

#### Accessibility by downstream applications

To facilitate their adoption, pangenome representations should be easily processed by downstream analyses. De Bruijn graphs are challenging to analyze due to their high number of nodes, edges, and redundancy (the $k-1$-overlaps between nodes). Though De Bruijn graph representations usually support queries of presence/absence on nodes (as in Bifrost), they lack tools able to perform more elaborate analyses such as those discussed in the “Interpretation of variation in pangenome graphs: focus on two HLA loci” section, e.g., incorporating haplotype information at the *k*-mer level. On the other hand, variations graphs with paths provide more flexibility, i.e., as in pggb and Minigraph-Cactus with the odgi visualization toolkit. Finally, in Minigraph, which considers a narrower spectrum of variants, the absence of path information prevents haplotype-level analysis; haplotypes would need to be manually mapped back to the graph. The choice of the pangenome building tool depends on the envisioned application. pggb and Minigraph-Cactus graphs have been shown to outperform linear references for short-read mapping, genotyping, and RNA sequencing mapping [8]. As these two methods are complex pipelines based on multiple tools where parameters have been carefully set, they can be more challenging to install and run than single integrated tools. Minigraph alone can also be used if the focus is on structural variation instead of SNPs or small indels, and to quickly produce a pangenome graph for complex loci visualization and interpretation. The dBG-based approaches show that, for example with Bifrost, they retain the same base-level information as more computational-heavy variation graph approaches, but the lack of tools to use them for analysis limits their adoption.

#### Haplotype compression

Building a graph pangenome can be seen also as a way to store, compact and retrieve the input haplotypes. As the number of new assemblies is growing faster than the data storing capacity, pangenomes can potentially help save storage space. This is highlighted by the disk space reported in Table 1, which is consistently smaller than the sum of haplotype sizes for all methods and datasets.

In order to losslessly retrieve the input genomes from a pangenome, the representation has to store all variations from the original haplotype sequences as paths in the graph. pggb and Minigraph-Cactus fall into this category while the other three considered tools do not store paths, or do not consider all variations, thus they are lossy.

Of note, the GBZ tool [9] enables graph pangenomes that store paths in the GFA file format to be stored in a lossless compressed form. It uses a Graph Burrows-Wheeler transformation to compress the graph in a more efficient way than using gzip [9]. Using GBZ, the pangenomes generated by pggb and Minigraph-Cactus are losslessly compressed with space gains of 3.5–5×.

#### Ease of visualization

Visualizing large graphs which exceed hundreds of thousands of node is a challenge that exceeds the scope of pangenomics. The H104 pangenomes are difficult to visualize. Among the visualization tools considered by the Human Pangenome Reference consortium [6], only Bandage is able to display the Minigraph or mdbg H104 graphs, which contains a few million nodes. We reduced the number of nodes and edges of pggb, Minigraph-Cactus and Bifrost H10 graphs by collapsing isolated subgraphs representing SNPs or indels up to 10k bp (using the command gfatools asm -b 10000 -u).

#### Quality of metadata and annotation

 Augmenting pangenome structures with information from other omics data would increase pangenome relevance in biological discoveries. As biobanks are rapidly growing, more data is available on regulatory regions, transcriptomics, CNVs and other medically relevant traits [22, 23]. Pangenome data structures could leverage such information and some of the considered tools offer basic functionality in this sense. Bifrost provides a function to link data to graph vertices through C++ APIs. pggb and Minigraph-Cactus, using odgi, offer annotation capabilities through insertion of paths or BED records. Minigraph and mdbg do not offer any annotation feature. Specifically, in order to enhance a pangenome graph with metadata (for example with genes and regulatory regions known variants), it is desirable to maintain compatibility with methods and data formats that use a linear reference. One could conceivably project data from a graph to a reference genome to continue downstream analyses using linear coordinates. A simple method to achieve this compatibility, in our view, is to store the reference genome of interest inside the graph pangenome that supports retrieving such a reference. Variation graphs built using pggb or Minigraph-Cactus, due to their locally acyclical and directed construction and their use of haplotype paths, store all the coordinates needed for such a task. Haplotype paths play an important role as they avoid additional mapping to the graph, by using the odgi tool to extract or inject the required information. Minigraph does not store haplotype paths and requires mapping sequences to the graph to restore haplotype information. On the other hand, De Bruijn graphs, using associated color data, can record the membership of k-mers to a reference sequence, yet one cannot fully reconstruct a haplotype unless k-mers positions are also stored.

**Table 2:** Relative strengths of five pangenome graph construction tools. Explanation of rows: (1) efficacy of construction algorithm, measuring wall-clock time; (2) degree to which variants (e.g., SNPs) are retained; (3) ability of a tool to perform well on large datasets, both in comparison to other tools but also compared to smaller datasets; (4) ability to modify the produced data structure to add or remove haplotypes; (5) property of producing the same result irrespective of perturbations, such as permutation of the input order, and repeating the same run; (6) existence of tools (and operations) that can be applied to the resulting graphs; (7) whether input haplotypes information is retained by the tools, and if so, its space efficiency; (8) whether the entire graph can be directly visualized and interpreted; (9) easiness of “zooming in” a specific genomic region and interpret variants; (10) summarizes the functionalities provided by the tools to annotate the pangenomes with genomic data; (11) ability to share information between the graph and a linear reference

Metric	Bifrost	pggb	Minigraph-Cactus	Minigraph	mdbg
1) Construction speed	$\bullet \bullet \circ$	$\bullet \circ \circ$	$\bullet \circ \circ$	$\bullet \bullet \circ$	$\bullet \bullet \bullet$
2) Variations	$\bullet \bullet \bullet$	$\bullet \bullet \bullet$	$\bullet \bullet \bullet$	$\bullet \bullet \circ$	$\bullet \bullet \circ$
3) Scalablilty	$\bullet \bullet \bullet$	$\bullet \circ \circ$	$\bullet \circ \circ$	$\bullet \bullet \circ$	$\bullet \bullet \bullet$
4) Editability	$\bullet \bullet \bullet$	$\bullet \bullet \circ$	$\bullet \circ \circ$	$\bullet \bullet \circ$	$\bullet \circ \circ$
5) Stability	$\bullet \bullet \bullet$	$\bullet \circ \circ$	$\bullet \circ \circ$	$\bullet \bullet \circ$	$\bullet \bullet \bullet$
6) Accessibility by downstream applications	$\bullet \circ \circ$	$\bullet \bullet \bullet$	$\bullet \bullet \bullet$	$\bullet \bullet \circ$	$\bullet \circ \circ$
7) Haplotype compression performance	$\bullet \bullet \circ$	$\bullet \bullet \bullet$	$\bullet \bullet \bullet$	$\bullet \circ \circ$	$\bullet \circ \circ$
8) Ease of visualization	$\bullet \circ \circ$	$\bullet \bullet \circ$	$\bullet \bullet \circ$	$\bullet \bullet \bullet$	$\bullet \bullet \bullet$
9) Loci visualization and interpretability	$\bullet \circ \circ$	$\bullet \bullet \circ$	$\bullet \bullet \bullet$	$\bullet \bullet \circ$	$\bullet \circ \circ$
10) Metadata and annotation	$\bullet \bullet \circ$	$\bullet \bullet \bullet$	$\bullet \bullet \circ$	$\bullet \circ \circ$	$\bullet \circ \circ$
11) Compatibility with a linear reference coordinates	$\bullet \circ \circ$	$\bullet \bullet \bullet$	$\bullet \bullet \bullet$	$\bullet \bullet \circ$	$\bullet \circ \circ$

## Discussion

Five state-of-the-art pangenome graphs construction tools were compared on the representation of up to 104 human haplotypes. The approaches significantly differ in terms of speed, graph size, and representation of variations. We find that it remains computationally prohibitive to generate human pangenome graphs for hundreds of haplotypes, especially while retaining all variations. Each approach has its own set of strengths, and ultimately the choice of the method depends on the downstream application. In addition, several takeaway points emerged from our analysis.

First, our focused analysis of HLA loci revealed that de Bruijn graphs and variation graphs represent genomic variations equally well as pangenomes. This is of particular importance as also shown by the draft human pangenome references [8]: pangenomes are pivotal to trace complex and clinically relevant loci. While de Bruijn graphs are faster to construct, more stable, and scale better in terms of input size, the resulting graphs are challenging to interpret downstream. Variations graphs on the other hand are more practical to analyze at the expense of a less efficient construction step. Their visualization are more straightforward to interpret, mostly due to not having cycles, and provide insights into loci differences.

Second, we can highlight two categories of pangenomic methods that have distinct application domains. pggb, Minigraph-Cactus and Bifrost store all possible variations, and keep haplotype information as paths or colors. They provide a complete picture of the set of variations in the input genomes which makes them difficult to analyze. They can be used for a large variety of genomic analysis, as shown for pggb and Minigraph-Cactus [8]. Minigraph and mdbg generate ’sketched’ pangenome graphs that consider only large variants, ignoring smaller differences, and are more efficient to construct and visualize. They can be used for large scale characterization of variation in population, as proven for bacteria [15].

Third, every tool possesses an exclusive set of features. pggb facilitates downstream analyses using the companion tool odgi. It allows to precisely extract and browse any locus of interest. It is the only tool that generates variation graphs without a reference. It also keeps a lossless representation of the input sequences. Minigraph generates a pangenome graph based on a reference sequence taken as a backbone. It shines in the representation of complex structural variations, but does not include small or inter-chromosomal variations. The pipeline Minigraph-Cactus, which uses the Cactus base aligner, can be used to add small-level variations on top of the Minigraph graph and to keep a lossless representation of the input sequences. Bifrost illustrates that classical de Bruijn graphs are scalable, stable, dynamic, and store all variations. However, extracting information from them remains a challenge. Lastly, mdbg is the fastest construction method which generates an approximate representation of differences between haplotypes. As discussed in the “Accessibility by downstream applications” section, these features enable different genomic analyses and downstream applications.

## Conclusions

In conclusion, our results highlight the strengths and weaknesses of current pangenome construction tools for human applications, with a specific focus on how do they represent specific loci of medical relevance. We also provide insights on the features they possess and point out their best application domains. In our view, future directions for human pangenomes building tools should focus on tackling efficiency bottlenecks, aiming to represent hundreds to thousands of haplotypes. Representations should further be lossless and represent the input haplotypes as paths in the graph. Such features would unlock many other applications such as lossless compression of haplotypes and cancer copy number variant analysis. Finally, we recognize the need for more user-friendly tools that can be used by biologists and that can translate complicated questions into graph queries. While odgi begins to address these questions in variation graphs, other approaches have not yet provided user-friendly interfaces. A package similar to odgi for de Bruijn graphs would help fully realize their potential.

## Methods

### Datasets and haplotypes collection

In order to evaluate the state of current human pangenome representations, we sought to build a human pangenome that contains all publicly available high-quality human haplotypes. We collected from two different sources 102 different haplotypes from the genome of 51 individuals, and also used the two reference genomes, GRCh38 from the Genome Reference Consortium (GRC) [24] and CHM13 v2.0 cell line of the T2T Consortium [25]. Five haplotypes correspond to Google Brain Genomics DeepConsensus [26] assembly dataset: they are hifiasm assemblies of PacBio Hi-Fi reads corrected with DeepConsensus. The average of their N50 is 37.99 Mbp. The remaining haplotype assemblies as well as the T2T reference are from the Human Pangenome Reference Consortium (HPRC) year-1 freeze [6], and GRCh38 is from the GRC. Their average N50 is 40.3 Mbp. Since HG002 is contained in the DeepConsensus data, the HPRC HG002 haplotypes were not used. The origin and the sex of the individuals are diverse and provide a fair representation of the diversity in human population: out of 51 total individuals, 21 are males and 30 are females and they represent 14 different ethnic groups, from USA to Africa and Asia. We did not perform any additional selection, regarding sex and ethnicity, on these public datasets as our main goal was to use as many genomes as possible. However, the HPRC stated that the genomes were selected to represent genetic diversity in humans [8]

To evaluate the scalability of pangenome construction tools, we created three datasets of increasing size: (1) 2 haplotypes from the same individual, HG006; (2) 10 haplotypes from 5 different individuals (HG002, HG003, HG004, HG006, and HG00735); and finally, (3) all of the 104 haplotypes. To test whether the order of the input sequences matters, we considered various random orderings for the 10 haplotypes in Dataset 2. Since Minigraph needs a reference sequence as the first haplotype in order to correctly build the graph, we generated specific 2 and 10 haplotype datasets with the first haplotype replaced by the reference genome CHM13. This was applied to the Minigraph-Cactus pipeline as well as it uses Minigraph variation graphs (Table 3).

**Table 3:** Description of the three datasets generated to test the scalability of the tools. Data sources: ^1^ Google Brain Genomics [27]; ^2^ Human Pangenome Reference Consortium [28]; ^3^ 1000 Genomes Project [28]; ^4^ Telomere to Telomere Consortium [28]

Haplotypes	Project	Bases
2	Google^1^	5.9 Gbp
10	Google, HPRC^2^	30 Gbp
104	Google, HPRC, 1KG^3^, T2T^4^	313.6 Gbp

### Pangenome graph construction tools

We evaluated tools that generate graph pangenomes as variation graphs and colored compacted de Bruijn graphs. Variation graphs are generally locally acyclic while de Bruijn graphs have cycles. In variation graphs, nodes represent sequences and edges represent immediate sequence adjacency without overlap. Variation graphs are generally easier to visualize and to interpret while challenging to construct at scale and, apart from pggb, require a reference genome. In de Bruijn graphs (dBG), nodes are *k*-mers (string of length k) and edges are (k-1)-overlaps between nodes. In practice, dBGs are represented in a compact way where all nodes along unbranching paths are compacted into *unitigs*. The resulting graph is called compacted De Bruijn Graph, where nodes are unitigs and edges represent (k-1)-overlaps. As shown in Fig. 1, de Bruijn graphs result in large graphs that pose visualization and interpretation challenges, in particular as there is no alignment to a reference (Table 4).

- Bifrost constructs dynamic, colored compacted de Bruijn Graphs (*cdBG*). It first generates a standard dBG using an efficient variant of Bloom Filters and then computes the compacted dBG from it. Colors, i.e., identifiers representing the sample origin of each k-mer are added by storing an array per *k*-mer. A human genome cdBG typically consists of a single large connected component, as common *k*-mers are shared between chromosomes. This pangenome representation contains all the variations present in input sequences.
- mdbg builds a variant of de Bruijn graphs called a minimizer-space de Bruijn Graph (mdbg), which is efficient to construct as it only considers a small fraction of the input nucleotides. Color information is currently not supported in the implementation. Similarly to Bifrost, a mdbg also typically represents a human genome as a single large connected component, albeit with orders of magnitude less nodes. Minimizer-space de Bruijn graphs mostly discard small variants, yet are sensitive to heterozygosity which creates branches in the graph.
- Minigraph constructs a directed, bidirected and acyclic variation graph iteratively by mapping new haplotypes using a combination of the minimap2 tool and the graph wavefront alignment algorithm. The first input sequence acts as a backbone for the whole representation. The sample(s) of each node are stored in a rGFA output file. Minigraph considers only variations longer than 50 bps hence it is oblivious to isolated SNPs and small indels: even if it produces base-level alignment for contigs, the graphs are not a base-level resolution. The resulting graph is divided into connected components that correspond to the chromosomes present in the first given input genome.
- Minigraph-Cactus is a variation graph construction pipeline that combines Minigraph to generate a structural variations graph and Cactus base aligner to generate base-level pangenome graphs of a set of input assemblies and embed haplotypes paths. Cactus [16] is a highly accurate and scalable reference-free multiple whole-genome alignment tool, that in this pipeline considers the reference sequence used by Minigraph to ensure that the resulting variation graph is acyclic. The final graph is further normalized using GFAffix[29]. The pipeline allows to generate multiple graphs, one for each chromosome, or produce a single graph that includes inter-chromosomal variants.
- pggb is a directed acyclic variation graph construction pipeline rather than a single tool. It calls three different tools: pairwise base-level alignment of haplotypes using wfmash [30], graph construction from the alignments with seqwish [31], graph sorting and normalization with smoothxg and GFAffix [29, 32]. The resulting variation graph represents variations of all lengths present in the input sequences.

**Table 4:** URL, version, pangenome representation, and parameters of the three analyzed tools. pggb/0.2.0 used wfmash v0.7.0, seqwish v0.7.3, and smoothxg v0.6.1

Tool	Github repository	Graph type	Version	Parameters
Bifrost	pmelsted/Bifrost	De Bruijn graph	1.0.5	-k100 -c
pggb	pangenome/pggb	variation graph	0.2.0	-p 98 -s 10000 -k 311 -G 13033,13117
				-O 0.03 -v -t 8 -T 8 -A -Z
Minigraph	lh3/Minigraph	variation graph	0.18	-cxggs
Minigraph-Cactus	ComparativeGenomics	variation graph	2.2.3	–maxLen 10000 –delFilter 10000000
	Toolkit/cactus			
mdbg	ekimb/rust-mdbg	De Bruijn graph	1.0.1	-k 10 -d 0.0001 –minabund 1 –reference

### Supplementary Information

**Additional file 1.** Review history.

## Data Availability

The scripts used to generate and analyze the pangenomes can be found at [35][36] under MIT license. Google Brain Genomic assemblies can be found at [27]. HPRC assemblies, CHM13 and GRCh38 can be found at [28].

## Appendix

### Benchmark infrastructure

Running time of pangenome construction tools was measured as wall clock time and peak memory as maximum resident set size using the time command. Other metrics were obtained with custom Python scripts. All benchmarks were performed on a Supermicro Superserver SYS-2049U-TR4, with 3 TB RAM and 4 Intel SKL 6132 14-cores @ 2.6 GHz, using 8 cores.

### TwoPaCo

We did not consider TwoPaCo as it is redundant with Bifrost. Both methods construct the same de Bruijn graphs. TwoPaCo is a method for constructing ccdBG by finding junction *k*-mers at the boundaries of unitigs or in branching nodes. It consists of two main steps in which it approximates the dBG with a Bloom filter in order to reduce the size of the problem and then runs a two pass highly parallel algorithm on it. It constructs ccdBGs similarly to Bifrost. Bifrost is faster, supports edit operations, and accepts also reads other than assemblies as input. We tested both tools on NCBI datasets from three different known human variation regions part of the human leukocyte antigen (HLA) complex: HLA-A, MICB and TAP1. These loci have different number of sequences and have complexity and length. The resulting graphs have exactly the same k-mer content and substantially equal topology. The difference is that TwoPaCo considers sequences with IUPAC ’N’ bases while Bifrost does not and that in some cases TwoPaCo renders some unitigs split in two or more consecutive nodes.

### Loci extraction method

For Bifrost and mdbg graphs, nodes corresponding to the input sequences are identified with GraphAligner [33] and the subgraph is extracted using the Bandage *reduce* function. As the aligned nodes are not expected to represent the full diversity of the population in the pangenomes, the considered portion of the graph contains also nodes up to a certain distance from the aligned ones: 1 for mdbg and 3 for Bifrost. This number is based on the size of the sequences spelled by the nodes and on the considered variations. Artifacts, mostly tips, that are not part of the locus of interest are removed with a custom python script. For Minigraph generated graphs, the Minigraph own alignment function has been used to identify the nodes and then Bandage is used to extract the subgraph. For pggb, first we generate a bed file of the position of the region of interest in every haplotype used to construct the graph. The ranges are derived from aligning them to the locus sequence(s) using minimap2 [34]. The graph corresponding to the region is then extracted using the odgi extract and odgi view functions. For Minigraph-Cactus we use the same strategy as pggb, with the difference that the bed file is only for the reference CHM13, present in the graph. The annotation of the specific loci in the subgraph is done using nodes from the alignment with Minigraph or GraphAligner to the subgraph. This makes it possible to highlight multiple sections in the region, as, for example, genes and pseudogenes of interest.

## References

[1] Haeussler M, Zweig AS, Tyner C, Speir ML, Rosenbloom KR, Raney BJ (2019). The UCSC genome browser database: 2019 update. Nucleic Acids Res..

[2] Garrison E, Sirén J, Novak AM, Hickey G, Eizenga JM, Dawson ET (2018). Variation graph toolkit improves read mapping by representing genetic variation in the reference. Nat Biotechnol..

[3] Consortium TCPG (2016). Computational pan-genomics: status, promises and challenges. Brief Bioinforma..

[4] Sirén J, Monlong J, Chang X, Novak AM, Eizenga JM, Markello C (2021). Pangenomics enables genotyping of known structural variants in 5202 diverse genomes. Science..

[5] Sherman RM, Salzberg SL. Pan-genomics in the human genome era. Nat Rev. 2020;Genet(21):243–254. 10.1038/s41576-020-0210-7. 10.1038/s41576-020-0210-7 PMC7752153 32034321

[6] Wang T, Antonacci-Fulton L, Howe K, Lawson HA, Lucas JK, Phillippy AM (2022). The Human Pangenome Project: a global resource to map genomic diversity. Nature..

[7] Ebler J, Ebert P, Clarke WE, Rausch T, Audano PA, Houwaart T (2022). Pangenome-based genome inference allows efficient and accurate genotyping across a wide spectrum of variant classes. Nat Genet..

[8] Liao WW, Asri M, Ebler J, Doerr D, Haukness M, Hickey G (2023). A draft human pangenome reference. Nature..

[9] Sirén J, Paten B (2022). GBZ file format for pangenome graphs. Bioinformatics..

[10] Sheikhizadeh S, Schranz ME, Akdel M, de Ridder D, Smit S (2016). PanTools: representation, storage and exploration of pan-genomic data. Bioinformatics..

[11] Holley G, Melsted P (2020). Bifrost: highly parallel construction and indexing of colored and compacted de Bruijn graphs. Genome Biol..

[12] Garrison E, Guarracino A, Heumos S, Villani F, Bao Z, Tattini L, et al. Building pangenome graphs bioRxiv. 2023. 10.1101/2023.04.05.535718.

[13] Minkin I, Pham S, Medvedev P (2016). TwoPaCo: an efficient algorithm to build the compacted de Bruijn graph from many complete genomes. Bioinformatics..

[14] Li H, Feng X, Chu C (2020). The design and construction of reference pangenome graphs with minigraph. Genome Biol..

[15] Ekim B, Berger B, Chikhi R (2021). Minimizer-space de Bruijn graphs: Whole-genome assembly of long reads in minutes on a personal computer. Cell Syst..

[16] Armstrong J, Hickey G, Diekhans M, Fiddes IT, Novak AM, Deran A (2020). Progressive Cactus is a multiple-genome aligner for the thousand-genome era. Nature..

[17] Hickey G, Monlong J, Ebler J, Novak AM, Eizenga JM, Gao Y, et al. Pangenome graph construction from genome alignments with Minigraph-Cactus. Nat Biotechnol. 2023. 10.1038/s41587-023-01793-w. 10.1038/s41587-023-01793-w PMC10638906 37165083

[18] Chin CS, Behera S, Metcalf G, Gibbs RA, Boerwinkle E, Sedlazeck FJ. A pan-genome approach to decipher variants in the highly complex tandem repeat of LPA. bioRxiv. 2022. 10.1101/2022.06.08.495395.

[19] Dendrou CA, Petersen J, Rossjohn J, Fugger L (2018). HLA variation and disease. Nat Rev Immunol..

[20] Vietzen H, Zoufaly A, Traugott M (2021). Deletion of the NKG2C receptor encoding KLRC2 gene and HLA-E variants are risk factors for severe COVID-19. Genet Med..

[21] Guarracino A, Heumos S, Nahnsen S, Prins P, Garrison E. ODGI: understanding pangenome graphs. Bioinformatics. 2022;Btac308. 10.1093/bioinformatics/btac308. 10.1093/bioinformatics/btac308 PMC9237687 35552372

[22] 100,000 Genomes Pilot on Rare-Disease Diagnosis in Health Care — Preliminary Report. N Engl J Med. 2021;385(20):1868–1880. 10.1056/NEJMoa2035790. 10.1056/NEJMoa2035790 PMC7613219 34758253

[23] Johnson R, Ding Y, Venkateswaran V, Bhattacharya A, Boulier K, Chiu A (2022). Leveraging genomic diversity for discovery in an electronic health record linked biobank: the UCLA ATLAS Community Health Initiative. Genome Med..

[24] Schneider VA, Graves-Lindsay T, Howe K, Bouk N, Chen HC, Kitts PA (2017). Evaluation of GRCh38 and de novo haploid genome assemblies demonstrates the enduring quality of the reference assembly. Genome Res..

[25] Nurk S, Koren S, Rhie A, Rautiainen M, Bzikadze AV, Mikheenko A (2022). The complete sequence of a human genome. Science..

[26] Baid G, Cook DE, Shafin K, Yun T, Llinares-López F, Berthet Q, et al. DeepConsensus improves the accuracy of sequences with a gap-aware sequence transformer. Nat Biotechnol. 2022. 10.1038/s41587-022-01435-7. 10.1038/s41587-022-01435-7 36050551

[27] Baid G, Cook DE, Shafin K, Yun T, Llinares-López F, Berthet Q, et al. Dataset. Google Brain Assemblies. 2023. https://console.cloud.google.com/storage/browser/brain-genomics-public/research/deepconsensus/publication/analysis/genome_assembly. Accessed 15 Dec 2022.

[28] Liao WW, Asri M, Ebler J, Doerr D, Haukness M, Hickey G, et al. Dataset. Human Pangenome Reference Consortium Asseblies. 2023. https://s3-us-west-2.amazonaws.com/human-pangenomics/index.html?prefix=working/. Accessed 15 Dec 2022.

[29] Doerr D. Gfaffix identifies walk-preserving shared affixes in variation graphs and collapses them into a non- redundant graph structure. 2021. https://github.com/marschall-lab/GFAffix. Accessed Aug 2022.

[30] Guarracino A, Mwaniki N, Marco-Sola S, Garrison E. wfmash: whole-chromosome pairwise alignment using the hierarchical wavefront algorithm. 2021. https://github.com/ekg/wfmash. Accessed 15 Dec 2022.

[31] Garrison E, Guarracino A. Unbiased pangenome graphs. Bioinformatics. 2022;39(1):btac743. 10.1093/bioinformatics/btac743. 10.1093/bioinformatics/btac743 PMC9805579 36448683

[32] Guarracino A, Garrison E. smoothxg: local reconstruction of variation graphs using partial order alignment. 2021. https://github.com/pangenome/smoothxg. Accessed 15 Dec 2022.

[33] Rautiainen M, Marschall T (2020). GraphAligner: rapid and versatile sequence-to-graph alignment. Genome Biol..

[34] Li H (2018). Minimap2: pairwise alignment for nucleotide sequences. Bioinformatics..

[35] Andreace F. Github sourcecode. 2023. https://github.com/frankandreace/CRHPG. Accessed 15 Oct 2023.

[36] Andreace F (2023). Zenodo sourcecode..

