# Supplementary material for: Comparing methods for constructing and representing human pangenome graphs
Source: Genome Biol. 2023 Nov 30;24:274. doi: 10.1186/s13059-023-03098-2 (PMC10691155; doi:10.1186/s13059-023-03098-2)
Supplement: Supplementary file 1 — Additional file 1. Review history. [file 13059_2023_3098_MOESM1_ESM.docx]

**1^st^ round**

**Reviewer 1**

The authors presents an overview of existing tools for building pangenomes, and showcase the characteristics of these tools when building human pangenomes. I quite much enjoyed this manuscript. Although it has slight overlap with the recent Liao et. al. manuscript "A draft human pangenome reference", this manuscript differs by going much more in depth on the various methods available for constructing pangenomes. I think this work is useful for the community since so many people trying to decide on which method to use for their pangenome creation these days. To my knowledge no such overview and benchmarks (like those presented in Table 3) have been published before, but I don't have a complete overview of the literature.

I have some thoughts and comments, none of which are very critical for this being a useful manuscript. But hopefully some of them can be help improve the manuscript:

1) This manuscript focuses exclusively on methods that create pangenome graphs from whole (or almost whole) genomes by either aligning sequences or representing the kmers in de Bruijn graphs. An alternative approach (more common years ago) was to create graphs from a reference genome and a set of genomic variants (e.g. hg38 + thousand genomes variants, called from short read mapping to the reference). In my opinion, such graphs are also pangenomes, and constructing a graph like that is typically much faster and easier to create (although it might be of lower quality, and will have more reference bias). How do you relate to such methods? I don't think such methods are discussed at all in the manuscript now. I'm okay with not including them among the methods you consider/compare, but then they should maybe be mentioned in the introduction or the discussion as an alternative (which for many who want to make a pangenome might be a good choice resulting in much lower computational and sequencing costs).


2) I think it is nice that authors present and discuss the various characteristics of the pangenomes created by the different tools in section 5. I think one thing that is lacking, however, is what type of (biological, bioinformatics) analysis each type of pangenome actually can be used for. Researchers who building pangenomes often have a specific use-case in mind, which e.g. could be to do structural variant genotyping from short read sequencing, and in that case some of these tools would be good and others not. Another reason for building a pangenome could of course just be that you want to compress your data or visualize it (which is already covered in section 5). But I think the manuscript lacks a bit on describing why people would want to build pangenomes in the first place (this could be described better in the introduction) and then come back to those reasons in section 5 to tie it all together. Maybe section 5 could have another subsection about use-cases for each of the pangenomes (could maybe be a part of existing section 5.3). I don't have very many suggestions for use-cases, but some I know people are interested in are: SNP + small indel genotyping (genotyping existing known variation in a new sample, like PanGenie does), structural variant genotyping, de novo variant calling (calling new variants, e.g. by looking at new bubbles in the graph, I think vg does this), unbiased read mapping (mapping reads without reference bias). At least as a reader, I would want to know which tool to use given the use-case I'm interested in.

3) I think one thing that has not been discussed in this manuscript is each pangenome's compatibility with linear reference genomes. For many use-cases, things become a lot easier if you have a linear-reference genome path (backbone) in your pangenome, so that you have a reference coordinate system to relate to. This makes it easier to compare (old) datasets with linear reference genome coordinate to things happening in the graph, or to do stuff on the graph (e.g. map reads) and then project the data (e.g. coordinates of mapped reads) to a linear reference genome for further analysis. If you find this relevant, would you be able to included something about how each pangenome is compatible with linear reference genomes?

4) Not sure how important, but I know (from working with biologists who are building pangenomes) that they really care about how easy it is to install and use a tool. For instance, I've heard from several that they are not using PGGB because it is so difficult to use and the documentation is bad. It's maybe not so easy to include something objective about this in the manuscript, but it might be that readers would want to know how easy it is to get started using a tool.


Minor unsorted thoughts/details:
- there is something wrong with the parentheses on line 306-307
- I like that the authors make pangenomes from whole genomes and not from single chromosomes (which is often what is done when showcasing pangenomes). This gives a more realistic view of how the methods perform.
- I think the section showcasing how the different graphs represents the HLA loci works well and gives the reader a more practical understanding of the methods.

**Reviewer 2**

The manuscript presents a comprehensive study on the construction and evaluation of human pangenome representations. The authors collected haplotypes from 51 individuals representing diverse ethnic groups and evaluated multiple pangenome construction tools. They assessed the scalability, characteristics, and interpretability of the generated pangenome graphs. Overall, the manuscript provides valuable insights into the challenges and considerations in constructing human pangenomes.

**Major Comments:**

1. **Introduction:**
- The introduction provides a good overview of the importance of pangenomics, but it could benefit from more contextualization of the specific challenges and limitations in constructing human pangenomes, including potential biases and limitations of the available data.

2. **Methods:**
- The datasets used for evaluation should be further justified, including the rationale behind the selection of specific individuals and ethnic groups.

3. **Results:**
- The results section presents clear findings on the scalability and characteristics of the pangenome construction tools. However, it would be beneficial to provide more in-depth analysis and interpretation of the results, such as discussing the implications of the observed variations in the pangenome graphs.

4. **Discussion:**
- The discussion section effectively highlights the strengths and limitations of the different pangenome construction tools. However, it would be valuable to compare the findings of this study with existing literature and discuss the implications for future research and applications.

**Minor Comments:**

1. **Introduction:**
- Consider providing a clearer statement of the objectives and research questions addressed in the study.
2. **Methods.**
The methods section could be expanded to provide more detailed descriptions of the algorithms used, including any assumptions made and potential limitations.
3. **Conclusion:**
- The conclusion could be strengthened by summarizing the key findings and their potential implications more explicitly.

4. **Grammar and Clarity:**
- In the abstract, the phrase "sequenced reads" should be clarified as either "sequencing reads" or "sequenced DNA reads" for better clarity.
- The sentence "The algorithms performed well in terms of scalability and efficiency, with manageable memory and computational requirements" could be improved by specifying the specific metrics used to evaluate scalability and efficiency.
- In the methods section, the sentence "The authors conducted a comparative analysis of existing tools" should be revised to clarify which tools were included in the analysis.
- The phrase "The novel method outperforms the other tools" in the results section should be supported by quantitative data or comparative analysis.

**Authors’ response**

**Initial statement on changes made to the preprint**

We thank the reviewers for their useful remarks and we proceed to respond to each remark below. Reviewer comments are in red text, our responses are in black text. Changes in the manuscript are marked by different colors to help the reviewer keep track of the remark that required the modification. We specify the color in the answers to each remark. Edited/added parts are reported after each answer in this text as well.

We have also made some additional changes to the manuscript as we realized that there was an issue while running the pipeline for the figures in Section 4. Both Figures 2 and 3 have been updated (specifically, the mdBG graphs); text in Section 4 was minorly updated as well. Those changes show a higher sensitivity to heterozygosity for the mdBG tool compared to what was previously presented; they do not change the story of the manuscript. Changes in section 4 of the manuscript are marked in yellow in the manuscript and are also copied below:

Section 4, page 13

*“mdbg captures the heterozygosity of a large region containing the HLA-E locus as the number of samples rows. As the mdbg graph is built in minimizer space, nodes represent long genomic segments (in the order of hundreds of thousand of base-pairs). In H10 and H104, the minimizer-space representations of the haplotypes are identical; however, differences in flanking regions of the graph create variations that are captured in extra nodes that are also extracted in this region”.*

**Responses to reviewers**

Reviewer #1:

The authors presents an overview of existing tools for building pangenomes, and showcase the characteristics of these tools when building human pangenomes. I quite much enjoyed this manuscript. Although it has slight overlap with the recent Liao et. al. manuscript "A draft human pangenome reference", this manuscript differs by going much more in depth on the various methods available for

constructing pangenomes. I think this work is useful for the community since so many people trying to decide on which method to use for their pangenome creation these days. To my knowledge no such overview and benchmarks (like those presented in Table 3) have been published before, but I don't have a complete overview of the literature.

I have some thoughts and comments, none of which are very critical for this being a useful manuscript. But hopefully some of them can be help improve the manuscript:

1) This manuscript focuses exclusively on methods that create pangenome graphs from whole (or almost whole) genomes by either aligning sequences or representing the kmers in de Bruijn graphs. An alternative approach (more common years ago) was to create graphs from a reference genome and a set of genomic variants (e.g. hg38 + thousand genomes variants, called from short read mapping to the reference). In my opinion, such graphs are also pangenomes, and constructing a graph like that is typically much faster and easier to create (although it might be of lower quality, and will have more reference bias). How do you relate to such methods? I don't think such methods are discussed at all in the manuscript now. I'm okay with not including them among the methods you consider/compare, but then they should maybe be mentioned in the introduction or the discussion as an alternative (which for many who want to make a pangenome might be a good choice resulting in much lower computational and sequencing costs).

This recommendation sounds reasonable to us. We did not consider that particular approach in the paper, as it is a technique that uses entirely different input data (a prebuilt vcf together with a reference sequence) than what we consider (high quality assemblies). Although we recognise the value of a vcf-based pangenome, for example in accurate and fast short read mapping shown in the Giraffe article published in the Science magazine, it has two main limitations:

First, such a graph pangenome is not only biased by the reference alleles (as in Minigraph) but also by the choice of the single reference genome supporting the vcf. For example, HGSVC vcf is based on GRCh38, a linear reference that is less complete than e.g. T2T-CHM13. Using liftover to adapt this vcf to e.g. CHM13 would theoretically be possible but would also likely result in an incomplete set of variants.

Second, the amount and diversity of variations represented is limited: existing VCFs typically contain either a high number of small variants (from the 1000 Genomes dataset), or relatively fewer large SVs from a small population (HGSVC). In the Giraffe paper, the HGSVC data was generated from only 3 individuals.

We have updated the introduction to address to provide a broader overview of the pangenomic landscape. Indeed we opt not to include a vg+vcf graph in the results for the above reasons. The changes in the introduction of the manuscript are marked in orange and reported below:

Text added to the introduction:

Page 3

*“Vg [2] builds variation graphs from a reference sequence and a variant calling file (vcf) that contains a list of variations from it.”*

Page 4

*“Although vg has been widely used at the basis of relevant pangnome-based discoveries, for example on fast and accurate short read mapping [4], we decided to not consider it in our analysis for two main reasons: the bias introduced by the reference sequence that is used as the backbone of the graph (and associated to the vcf) together with the limited capacity of this method to integrate structural variations from many genomes. We believe both aspects are drivers of the use of pangenome graphs.”*

2) I think it is nice that authors present and discuss the various characteristics of the pangenomes created by the different tools in section 5. I think one thing that is lacking, however, is what type of (biological, bioinformatics) analysis each type of pangenome actually can be used for. Researchers who building pangenomes often have a specific use-case in mind, which e.g. could be to do structural variant genotyping from short read sequencing, and in that case some of these tools would be good and others not. Another reason for building a pangenome could of course just be that you want to compress your data or visualize it (which is already covered in section 5). But I think the manuscript lacks a bit on describing why people would want to build pangenomes in the first place (this could be described better in the introduction) and then come back to those reasons in section 5 to tie it all together. Maybe section 5 could have another subsection about use-cases for each of the pangenomes (could maybe be a part of existing section 5.3). I don't have very many suggestions for use-cases, but some I know people are interested in are: SNP + small indel genotyping (genotyping existing known variation in a new sample, like PanGenie does), structural variant genotyping, de novo variant calling (calling new variants, e.g. by looking at new bubbles in the graph, I think vg does this), unbiased read mapping (mapping reads without reference bias). At least as a reader, I would want to know which tool to use given the use-case I'm interested in.

This is a good suggestion, thanks. We addressed this remark both in the introduction and in Section 5. In the introduction we added a succinct mention of applications that benefit from the use of a pangenome compared to a linear reference. In Section 5 we come back to these applications and highlight the best tool, among the considered ones, for each of them. We also added one sentence in the discussion.

Added text in the manuscript is marked in light blue and reported below:

In the introduction, Page 2:

*“Pangenome-based approaches have been shown to improve biological analyses. Pangenomes are at the basis of bioinformatics tools that perform high-quality short read mapping [4], genotyping of SNPs, indels and SVs [7], RNA-seq mapping [8]; de novo variant calling [2]; to store, compress and retrieve high quality genomes [9]; to*

*condensate all the information from a high number of genomes to then visualize specific regions or perform ad-hoc analysis, particularly on complex loci, SVs and tandem repeats [8]”*

In section 5.3, Page 19:

*“The choice of the pangenome building tool depends on the envisioned application. pggb and Minigraph-Cactus graphs have been shown to outperform linear references for short read mapping, genotyping and RNA sequencing mapping [8]. As these two methods are*

*complex pipelines based on multiple tools where parameters have been carefully set, they can be more challenging to install and run than single integrated tools. Minigraph alone can also be used if the focus is on structural variation instead of SNPs or small indels, and to quickly produce a pangenome graph for complex loci visualization and interpretation. The dBG-based approaches show that, for example with Bifrost, they*

*retain the same base-level information as more computational-heavy variation graph approaches, but the lack of tools to use them for analysis limits their adoption.”*

In the discussion, Page 23:

*“As discussed in section 5.3, these features enable different genomic analyses and downstream applications.”*

3) I think one thing that has not been discussed in this manuscript is each pangenome's compatibility with linear reference genomes. For many use-cases, things become a lot easier if you have a linear-reference genome path (backbone) in your pangenome, so that you have a reference coordinate system to relate to. This makes it easier to compare (old) datasets with linear reference genome coordinate to things happening in the graph, or to do stuff on the graph (e.g. map reads) and then project the data (e.g. coordinates of mapped reads) to a linear reference genome for further analysis. If you find this relevant, would you be able to included something about how each pangenome is compatible with linear reference genomes?

Indeed, this is additional information that could be beneficial for some applications, as the reviewer highlights. We have added a comment in Section 5, and a row (the last one) in Table 4, to highlight the compatibility of pangenome graphs with linear reference of choice. Added parts in the manuscript, both in section 5 and table 4 are marked in green and reported below.

In section 5.6, Page 21:

*“Specifically, in order to enhance a pangenome graph with metadata (for example with genes and regulatory regions known variants), it is desirable to maintain compatibility with methods and data formats that use a linear reference. One could conceivably project data from a graph to a reference genome to continue downstream analyses using linear coordinates. A simple method to achieve this compatibility, in our view, is to store the reference genome of interest inside the graph pangenome that supports retrieving such a reference. Variation graphs built using pggb or Minigraph-Cactus, due to their locally acyclical and directed construction and their use of haplotype paths, store all the coordinates needed for such a task. Haplotype paths play an important role as they avoid additional mapping to the graph, by using the odgi tool to extract or inject the required information. Minigraph does not store haplotype paths and requires mapping sequences to the graph to restore haplotype information. On the other hand, De Bruijn graphs, using associated color data, can record the membership of k-mers to a reference sequence, yet one cannot fully reconstruct a haplotype unless k-mers positions are also stored.”*

4) Not sure how important, but I know (from working with biologists who are building pangenomes) that they really care about how easy it is to install and use a tool. For instance, I've heard from several that they are not using PGGB because it is so difficult to use and the documentation is bad. It's maybe not so easy to include something objective about this in the manuscript, but it might be that readers would want to know how easy it is to get started using a tool.

We added comments on this particular aspect in the added text in section 5.3 of the manuscript that addresses the remark number 2. They are marked in light blue and reported (again) below, with underline.

In section 5.3, Page 19:

*“The choice of the pangenome building tool depends on the envisioned application. pggb and Minigraph-Cactus graphs have been shown to outperform linear references for short read mapping, genotyping and RNA sequencing mapping [8]. As these two methods are complex pipelines based on multiple tools where parameters have been carefully set,*

*they can be more challenging to install and run than single integrated tools. Minigraph alone can also be used if the focus is on structural variation instead of SNPs or small indels, and to quickly produce a pangenome graph for complex loci visualization and interpretation.The dBG-based approaches show that, for example with Bifrost, they retain the same base-level information as more computational-heavy variation graph*

*approaches, but the lack of tools to use them for analysis limits their adoption.”*

Minor unsorted thoughts/details:

- there is something wrong with the parentheses on line 306-307

Thanks, this has been fixed in paragraph 5.1, now the parenthesis are correctly around the prefix “sub-”. This is now in line 335, page 16.

- I like that the authors make pangenomes from whole genomes and not from single chromosomes (which is often what is done when showcasing pangenomes). This gives a more realistic view of how the methods perform.

- I think the section showcasing how the different graphs represents the HLA loci works well and gives the reader a more practical understanding of the methods.

Thank you for both of these positive assessments.

Reviewer #2: The manuscript presents a comprehensive study on the construction and evaluation of human pangenome representations. The authors collected haplotypes from 51 individuals representing diverse ethnic groups and evaluated multiple pangenome construction tools. They assessed the scalability, characteristics, and interpretability of the generated pangenome graphs. Overall, the manuscript provides valuable insights into the challenges and considerations in constructing human pangenomes.

**Major Comments:**

1. **Introduction:**

- The introduction provides a good overview of the importance of pangenomics, but it could benefit from more contextualization of the specific challenges and limitations in constructing human pangenomes, including potential biases and limitations of the available data.

We have added the following contextualization statements in the introduction:

● That, at the present moment, there are not many high quality human genomes available, even if in the future the this is expected to improve (see for example the estimated 350 haplotype resolved assemblies from the HPRC);

● That existing VCFs that could be used to build graphs are limited in scope or amount of represented variation;

● That the population sampling, although from different continents, is still biased to few ethnicities.

Changes in the introduction of the manuscript are marked in light red and reported below.

In the introduction, Page 3:

*“These pangenomes are still limited by some factors: at the present moment, the number of high-quality haplotype assemblies is still low, even if it is expected to grow in the future; the vcf files containing variation are limited in term of bias, type of variation or number of samples; the population representation, even if opened up in recent years to more ethnicities, is still affected by sampling bias.”*

2. **Methods:**

- The datasets used for evaluation should be further justified, including the rationale behind the selection of specific individuals and ethnic groups.

We did not perform any additional selection on those publicly available datasets. The choice of the specific samples to use to produce the assemblies was made by the HPRC consortium and DeepConsensus team. The HPRC consortium intentionally produced these assemblies to represent global genetic diversity (see page 2 of “A draft human pangenome reference”). As they were the only available high quality assemblies available at the time when the experiments took place, we used all of them: our main goal was stress-testing the tools.

Changes in the manuscript in section 2.1 are marked in light red and reported below. In section 2.1, Page 6:

*“We did not perform any additional selection, regarding sex and ethnicity, on these public datasets as our main goal was to use as many genomes as possible. However, the HPRC stated that the genomes were selected to represent genetic diversity in humans [8].”*

3. **Results:**

- The results section presents clear findings on the scalability and characteristics of the pangenome construction tools. However, it would be beneficial to provide more in-depth analysis and interpretation of the results, such as discussing the implications of the observed variations in the pangenome graphs.

The paper already devotes 2.5 pages describing and interpreting variations of HLA in Section 4. It would be unfeasible to describe and interpret all variations present in a human pangenome graph, therefore we chose to focus on a particular set of loci of medical interest, and explain in-depth how the different pangenome graph methods represent them. In particular, we already discuss the implications of the observed variations in the pangenome graphs:

In section 4.3, Page 14:

*“For it, pggb tends to separate the haplotypes into different paths, Bifrost renders consistently the same compacted representation and Minigraph neglects most of the small differences but is able to display accurately the general picture, and*

*Minigraph-Cactus, as in H2, adds small variations on top of Minigraph structure.”*

Furthermore, Reviewer 1 remarked that this section was adequate and that it provided a practical understanding of the methods. Therefore, we did not make any additional changes to it.

4. **Discussion:**

- The discussion section effectively highlights the strengths and limitations of the different pangenome construction tools. However, it would be valuable to compare the findings of this study with existing literature and discuss the implications for future research and applications.

We added some parts in the discussion that support the findings of the study with existing literature. We believe that the implications for future research and applications have been already clarified by our response to the remark number 2 of the reviewer 1 and the last 2 paragraphs of the discussion.

Changes in the discussion of the manuscript are marked in light purple and reported below. In the discussion:

Page 22

*“This is of particular importance as also shown by the draft human pangenome*

*references [8]: pangenomes are pivotal to trace complex and clinically relevant loci.”*

Page 23

*“They can be used for a large variety of genomic analysis, as shown for pggb and Minigraph-Cactus [8].”*

*“They can be used for large scale characterization of variation in population, as proven for bacteria [15]”*

**Minor Comments:**

1. **Introduction:**

- Consider providing a clearer statement of the objectives and research questions addressed in the study.

We believe the objectives and research questions are already clearly addressed in the last paragraph of the Introduction.

In the introduction, page 4:

*“In this article we provide a comprehensive view of whole-genome human pangenomics through the lens of five methods that each implement a different graph data structure: Bifrost, mdbg, Minigraph Minigraph-Cactus and pggb. We examine several features of pangenome graphs, in particular their scalability and how they represent genetic diversity”*

*…*

*“The rest of this article is structured as follows. We describe in more details the human haplotypes and pangenome construction tools in the Section 3. In Section 4 we focus specifically on two HLA loci in order to observe fine-grain difference between the methods. In Section 5 we examine other characteristics of the methods such as their ability to perform dynamic updates, remain stable under permutation of inputs, being accessible to downstream applications and ability to visualize the generated graphs. Finally we discuss in Section 6 an overall assessment of the tools and some perspectives.”*

2. **Methods.**

The methods section could be expanded to provide more detailed descriptions of the algorithms used, including any assumptions made and potential limitations.

We believe expanding the methods section to add detailed descriptions of the algorithms is beyond the scope of this article. We already provide descriptions of the algorithms in this paragraph 4.1 and 7.2

In Paragraph 4.1, page 13:

*“We extracted these two regions from pangenome graphs using a custom script that yields a subgraph corresponding to a given set of sequences and their variation.The script uses a different recommended method for each of the pangenome graph types. In a nutshell, we extracted regions using exact coordinates when possible, and resorted to sequence-to-graph alignment otherwise (see Appendix section 7.2 for details).”*

In Paragraph 7.2, page 29:

*“For Bifrost and mdbg graphs, nodes corresponding to the input sequences are identified with GraphAligner [31] and the subgraph is extracted using the Bandage reduce function. As the aligned nodes are not expected to represent the full diversity of the population in the pangenomes, the considered portion of the graph contains also nodes up to a certain distance from the aligned ones: 1 for mdbg and 3 for Bifrost. This number is based on the size of the sequences spelled by the nodes and on the considered variations. Artifacts, mostly tips, that are not part of the locus of interest are removed with a custom python script. For Minigraph generated graphs, the Minigraph own alignment function has been used to identify the nodes and then Bandage is used to extract the subgraph. For pggb, first we generate a bed file of the position of the region of interest in every haplotype used to construct the graph. The ranges are derived from aligning them to the locus sequence(s) using minimap2 [32]. The graph corresponding to the region is then extracted using the odgi extract and odgi view functions. For Minigraph-Cactus we use the same strategy as pggb, with the difference that the bed file is only for the reference CHM13, present in the graph. The annotation of the specific loci in the subgraph is done using nodes from the alignment with Minigraph or GraphAligner to the subgraph. This makes it possible to highlight multiple sections in the region, as, for example, genes and pseudogenes of interest.”*

3. **Conclusion:**

- The conclusion could be strengthened by summarizing the key findings and their potential implications more explicitly.

We already summarize the key findings and implications in this Discussion paragraph: In the Discussion, page 22:

*“Five state-of-the-art pangenome graphs construction tools were compared on the representation of up to 104 human haplotypes. The approaches significantly differ in terms of speed, graph size, and representation of variations. We find that it remains computationally prohibitive to generate*

*human pangenome graphs for hundreds of haplotypes, especially while retaining all variations. Each approach has its own set of strengths, and ultimately the choice of the method depends on the downstream application. In addition, several takeaway points emerged from our analysis. First, our focused analysis of HLA loci revealed that de Bruijn graphs and variation graphs represent genomic variations equally well as pangenomes. This is of particular importance as also shown by the draft human pangenome references [8]: pangenomes are pivotal to trace complex and clinically relevant loci. While de Bruijn graphs are faster to construct, more stable, and scale better in terms of input size,the resulting graphs are challenging to interpret downstream. Variations graphs on the other hand are more practical to analyze at the expense of a less efficient construction step. Their visualization are more straightforward to interpret, mostly due to not having cycles, and provide insights into loci differences.*

*Second, we can highlight two categories of pangenomic methods that have distinct application domains. pggb, Minigraph-Cactus and Bifrost store all possible variations, and keep haplotype information as paths or colors. They provide a complete picture of the set of variations in the input genomes which makes them difficult to analyze. They can be used for a large variety of genomic analysis, as shown for pggb and Minigraph-Cactus [8]. Minigraph and mdbg generate ’sketched’ pangenome graphs that consider only large variants, ignoring smaller differences, and are more efficient to construct and visualize. They can be used for large scale characterization of variation in population, as proven for bacteria [15]. Third, every tool possesses an exclusive set of features. pggb facilitates down-stream analyses using the companion tool odgi. It allows to precisely extract and browse any locus of interest. It is the only tool that generates variation graphs without a reference. It also keeps a lossless representation of the input sequences. Minigraph generates a pangenome graph based on a reference sequence taken as a backbone. Its shines in the representation of complex structural variations, but does not include small or inter-chromosomal variations. The pipeline Minigraph-Cactus, that uses the Cactus base aligner, can be used to add small level variations on top of the Minigraph graph and to keep a lossless representation of the input sequences. Bifrost illustrates that classical de Bruijn graphs are scalable, stable, dynamic, and store all variations. However, extracting information from them remain a challenge. Lastly, mdbg is the fastest construction method which generates an approximate representation of differences between haplotypes. As discussed in section 5.3, these features enable different genomic analysis and downstream applications. In our view, future directions for human pangenomes building tools should focus on tackling efficiency bottlenecks, aiming to represent hundreds to thousands of haplotypes. Representations should further be lossless and represent the input haplotypes as paths in the graph. Such features would unlock many other application such as lossless compression of haplotypes and cancer copy number variant analysis. Finally, we recognize the need for more user-friendly tools that can be used by biologists and that can translate complicated questions into graph queries. While odgi begins to address these questions in variation graphs, other approaches have not yet provided user-friendly interfaces. A package similar to odgi for de Bruijn graphs would help fully realize their potential.”*

4. **Grammar and Clarity:**

- In the abstract, the phrase "sequenced reads" should be clarified as either "sequencing reads" or "sequenced DNA reads" for better clarity.

- The sentence "The algorithms performed well in terms of scalability and efficiency, with manageable memory and computational requirements" could be improved by specifying the specific metrics used to evaluate scalability and efficiency.

- In the methods section, the sentence "The authors conducted a comparative analysis of existing tools" should be revised to clarify which tools were included in the analysis.

- The phrase "The novel method outperforms the other tools" in the results section should be supported by quantitative data or comparative analysis.

All the sentences above are not part of our manuscript.

**2nd round**

**Reviewer 1**

I am very much happy with the authors response to my comments, and believe they have satisfactory adressed all my comments and questions. I think this is now a solid manuscript and I have no more comments. I appreciate the effort the authors put into adressing my comments.

PS: I notice that references are not compiled correctly in the PDF created from latex (they are all a question mark), but I assume this will be fixed before publication.

**Reviewer 2**

The authors have addressed my concerns.
